# The Relationship between Social Anxiety, Smartphone Use, Dispositional Trust, and Problematic Smartphone Use: A Moderated Mediation Model

**DOI:** 10.3390/ijerph18052452

**Published:** 2021-03-02

**Authors:** Anna Maria Annoni, Serena Petrocchi, Anne-Linda Camerini, Laura Marciano

**Affiliations:** 1 Institute of Public Health, Università della Svizzera italiana, 6900 Lugano, Switzerland; anne.linda.camerini@usi.ch; 2 Competence Centre on Ageing, Department of Business Economics, Health and Social Care, University of Applied Sciences and Arts of Southern Switzerland, 6928 Manno, Switzerland; 3 Faculty of Communication, Culture and Society, Università della Svizzera italiana, 6900 Lugano, Switzerland; serena.petrocchi@usi.ch (S.P.); laura.marciano@usi.ch (L.M.)

**Keywords:** problematic smartphone use, social anxiety, trust, moderation, mediation, young adults

## Abstract

Background: The pervasiveness of smartphones has raised concerns about an increase in the prevalence of problematic smartphone use (PSU), which depends on a set of psychological and behavioral risk factors. Previous research has yielded mixed results on factors predicting PSU, including social anxiety and trust. In particular, the role of trust remained largely unexplored. In the present study, we aimed to investigate the relationship between social anxiety and PSU, via the mediating role of time spent on the phone, and to explore the moderating role of dispositional trust toward others, by using a moderated mediation model with PSU as the outcome. Methods: A total of 240 young adults (M_age_ = 23.33, SD = 3.90, 50% male) answered an online questionnaire, which included the 12-item Social Anxiety Scale, a question on the daily duration of smartphone use, a single-item measure of dispositional trust, and the 10-item Smartphone Addiction Scale Short Version. Gender and occupational status were included as covariates. Results: Social anxiety was significantly and positively related to PSU; however, smartphone use did not mediate this relationship. Although the relationship between smartphone use and PSU was significant and positive, the link between social anxiety and smartphone use was not. Dispositional trust moderated and strengthened the direct relationships between social anxiety and PSU as well as smartphone use and PSU. Conclusions. Heavy smartphone users as well as socially anxious individuals, with the tendency of trusting others, are more at risk of PSU, which can be explained by their preference and search for online connections.

## 1. Introduction

The pervasiveness of the smartphone affects the private, social, and work lives, inasmuch as the constant presence of the device leads to a hyper-connected experience [1,2], where individuals are continuously connected to others and information through technological devices [3,4]. The widespread diffusion of the smartphone has solicited increased research interest in its use and problematic use, with specific attention paid to young adults [5,6,7]. In 2019, 96% of U.S. young adults owned a smartphone on which they spent, on average, 4 h per day. According to the same study, 15% of them could be classified as heavily attached to their device [8,9], which is an indicator of problematic smartphone use (PSU). PSU was found to be associated with different adverse outcomes, including sleep disorders [10,11], anxiety and depression [12,13], and decreased productivity and academic performances [14,15].

PSU shares some commonalities with the better-researched concept of internet use disorder (IUD) [16,17,18,19]. PSU usually involves: (i) spending a lot of time using the smartphone; (ii) unsuccessful attempts to reduce such behavior; (iii) reporting a positive mood when using it, while the mood changes to negative during offline periods; and (iv) reporting interpersonal problems (with family or friends, at work or school) due to excessive use [20]. It has been suggested that IUD should be considered as predominantly mobile or non-mobile, with PSU as an unspecified IUD form, predominantly mobile [19]. Similarly, gaming disorder [21], a condition warranting of further clinical attention and included in Section III of the fifth edition of the Diagnostic and Statistical Manual of Mental Disorders [22], also shares many characteristics with PSU and the suggested mobile/non-mobile taxonomy [19]. PSU has been described as leading to functional impairment and distress comparable to characteristics of other well-recognized (behavioral) addictions [6,23,24]. In general, the constant portability and connectivity of the device shapes a specific behavioral usage pattern that lays groundwork for smartphone-based behaviors and PSU to be considered as an umbrella category for both online and offline addictive activities [19].

To better understand the complexity of the causes and consequences of PSU, one can draw on the Interaction of Person-Affect-Cognition-Execution model (I-PACE) [25,26] model. The I-PACE is a comprehensive and multidisciplinary theoretical framework of new behavioral addictions; it foresees the integration of different research areas and current findings on internet-related disorders (e.g., PSU), including psychopathological features and dysfunctional personality traits and states. The I-PACE model suggests that personal characteristics, together with the contents of media consumption and the gratifications obtained, play a crucial role in developing PSU. There is evidence on an existing link between media addictions and dysfunctional psychological characteristics (the P-component), especially regarding the social sphere and, more precisely, social anxiety [27,28].

This evidence calls for a better understanding of the risk factors of PSU. These comprise, among others, personal and behavioral characteristics such as personality traits, anxiety levels, and the type and duration of smartphone use. In the present paper, we aim to examine the relationship between social anxiety and PSU, taking into consideration the mediating role of smartphone use and the moderating role of dispositional trust.

### 1.1. Social Anxiety and Problematic Smartphone Use

Besides the I-PACE model, Elhai and colleagues’ [29] model on anxiety symptoms and PSU conceptualizes anxiety as a risk factor of problematic smartphone use, through the mediation of anxiety-related transdiagnostic factors such as the fear of missing out or rumination and increased smartphone use. A meta-analysis reported a strong association between stress, general anxiety, and PSU [30]. Additionally, meta-analysis [31] has suggested that individuals with higher levels of neuroticism (a personality dimension determined by emotional instability, including high sensitivity to others’ critics, similar to social anxiety [32]), shows significant and positive associations with PSU and other problematic online activities. Although interesting, the abovementioned studies considered general anxiety symptomatology or personality traits in association with PSU. To date, literature on the role of *social* anxiety in predicting PSU is still limited. Social anxiety is defined by the fear of social scrutiny and negative evaluation, characterized by tension and nervousness in the social setting [33,34,35]. Individuals with higher levels of social anxiety tend to feel anxious about being disapproved or excluded by other people, and they are likely to interpret social stimuli as aversive or hostile [36]. An excessive amount of worry about social relationships can result in a pervasive fear of embarrassment accompanied by the avoidance of social or public situations, and, in extreme cases, social isolation [37]. Social anxiety is associated with a preoccupation for the “observer’s perspective” on the self [38]; therefore, by eliminating others’ face-to-face reactions of in-person interaction, the smartphone allows socially anxious individuals to find a safe place through which they can communicate [39]. One way to avoid offline social situations is by means of mediated communication and, more generally, smartphone use. More precisely, seeking relationships in the online environment may compensate for the lack of gratifying face-to-face relationships, which also explains the role of social anxiety in predicting internet addiction [40,41]. Based on past evidence, we formulated the following hypothesis:

**Hypothesis 1** **(H1).** *Higher levels of social anxiety are positively associated with higher levels of PSU*.

### 1.2. The Mediating Role of Smartphone Use

Smartphone use (SU) can be conceptualized in different ways, such as the duration or the frequency of use, the type of use, or the motivations and purposes of its use. These conceptualizations are not mutually exclusive, and past research focusing on the relationship with social anxiety has primarily used measures of time spent on the smartphone (duration) [21]. Although the time spent on the device is not problematic per se [42], an exaggerated amount of time spent on smartphones has been linked to PSU [43,44,45], and longitudinal studies have demonstrated that higher levels of SU predict PSU over time [46,47]. Hence, we decided to focus on the duration of smartphone use as one component of PSU.

Socially anxious individuals do not tolerate uncertainty well [48], and thus social interactions seek more frequently for reassurance. At the same time, as previously pointed out, they are more afraid of negative evaluations in face-to-face interactions. Lee and Stapinski [49] reported decreased social anxiety in online environments because they allow easier, less stressful, and more gratifying social interactions. This leads socially anxious people to check their smartphone more frequently in search for notifications [42,50], e.g., of incoming messages in online conversations. Previous studies demonstrated that the easy access to smartphone-mediated communication substitutes more anxious face-to-face social contacts [51,52] because it augments the sense of anonymity and diminishes appearance concerns [53], thus strengthening the relationship between social anxiety and the preference for mediated social interactions [49,54,55].

However, results from research are contradictory. A novel meta-analysis found a small-to-medium correlation between anxiety and smartphone use [30], whereas another meta-analysis [56] evidenced that social anxiety is not significantly related to time spent online, but to problematic internet use. In other studies, social anxiety was associated with more time spent on the device [39,57], e.g., on social networking sites [58]. Furthermore, heavy smartphone users reported higher levels of anxiety in cross-national samples [59]. Returning to Elhai and colleagues’ [29] model on anxiety symptoms and PSU, higher levels of smartphone use, both in terms of duration and frequency of use, are considered a mediator in the relationship between the two concepts. Following this model and empirical evidence supporting an association between social anxiety and increased smartphone use, we formulated the following hypothesis, concentrating on time spent on the device:

**Hypothesis 2** **(H2).** *Higher levels of social anxiety are positively associated with longer durations of smartphone use.*

Although smartphone use cannot be reduced to spending too much time with the device, the time spent on the smartphone has been considered an ubiquitous indicator [23,60] and predictor of PSU [45,46,47]. The I-PACE model also acknowledges media usage characteristics and, more precisely, a repeated use of digital devices for gratification needs as a risk factor of PSU [61]. Among young adults, smartphones are primarily used for communication purposes, through social media and instant messaging apps, indicating a predominant social interaction-driven usage [8]. Considering social smartphone use, Instagram, WhatsApp, and other social media platforms promote a prolonged and highly frequent use of the smartphone, with up to 74% of the respondents indicating to visit these platforms daily [9]. At the same time, non-social activities, such as gaming or viewing videos, have been linked to longer and more frequent use of the smartphone [62]. Past studies have shown that both social and non-social smartphone usage are associated with PSU [63,64,65]. Accordingly, there is consensus about a link between PSU and the excessive amount of time spent on the device [45], and longitudinal studies have demonstrated that higher levels of smartphone use predict PSU over time [46,47]. Thus, to complete our partial mediation model, where social anxiety is directly associated with PSU, and indirectly through the mediating role of smartphone use, we hypothesize that:

**Hypothesis 3** **(H3).** *A longer duration of smartphone use is positively associated with higher levels of PSU.*

### 1.3. The Moderating Role of Dispositional Trust

When it comes to communication and disclosure of information about the self in the online environment, a key personality factor to consider is dispositional trust. Dispositional trust is vital to healthy psychosocial functioning and a key variable for the formation and maintenance of healthy interpersonal relationships [66]. Dispositional trust can be described as a psychological state of voluntarily placing oneself in a vulnerable position based on confident expectations of the good intentions and actions of others [67].

Previous studies showed that the higher the trust, the greater the propensity to join online communications or actively seek for social interactions [68,69,70]. Dispositional trust motivates online communication intention, especially at the beginning of a relationship [71]. By increasing the intention to start an online interaction, dispositional trust increases people’s willingness to disclose and connect [71,72,73]. Accordingly, online trust was related to self-disclosure and reciprocity, which proved to positively influence social interactions [74,75]. Moreover, higher trust in others was associated with a greater amount of time spent online [76,77], especially in social media applications. This may reflect personal preferences towards online over face-to-face interactions, which can lead to more time spent on the device [61] and, if repeated habitually, to problematic device use [78]. High-trusting individuals also tend to be more positive and are more likely to accept new things, indicating a moderating role of dispositional trust in the online environment [79]. In the present study, we were interested in the moderating role of trust in the interplay between social anxiety and (problematic) smartphone use. More precisely, we expect a stronger association between smartphone use and PSU for individuals with higher levels of dispositional trust, especially considering the social use of the smartphone for communication and interaction purposes.

Furthermore, the moderating role of dispositional trust in reinforcing social anxiety and smartphone use, respective to PSU, needs further investigation. Higher levels of trust are likely to promote interaction seeking; however, combined with social anxiety, this would promote these interactions more in the online environment. As such, we can expect a stronger association between social anxiety and (problematic) smartphone use for individuals with higher trust levels. These considerations lead us to formulate the final hypothesis:

**Hypothesis 4** **(H4).** *Dispositional trust moderates the relationship between social anxiety, smartphone use, and PSU inasmuch as it strengthens the relationship among these concepts.*

**Figure 1:**
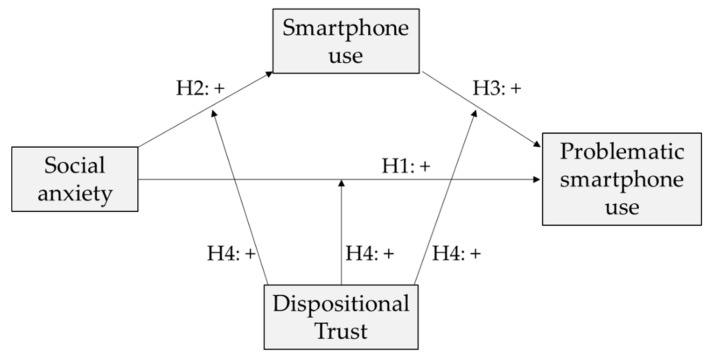
Theoretical moderated mediation model.

The theoretical moderated mediation model is represented in Figure 1.

## 2. Materials and Methods

### 2.1. Participants

The data for this study were collected during the baseline assessment of two experimental studies on smartphone-mediated communication conducted in spring and autumn 2019 [80]. Participants were recruited through flyers, Facebook advertisements, and snowball sampling in Canton Ticino (Switzerland). The experimental studies received approval from the Ethics Committee of the university where the research was carried out. After providing informed consent, participants filled out an online questionnaire with measures for the concepts of interest to this study alongside socio-demographic characteristics. There were no missing data due to the forced-answer format for all questions. The analytical sample consisted of 240 young adults (Mean age = 23.33, SD = 3.90; range 18–35 years old), balanced in gender (50% male, *n* = 120) and primarily based in Ticino, Switzerland (87.1%, *n* = 209). Most respondents were college students (62.9%, *n* = 151) or workers (32.9%, *n* = 79) and had graduated from college (36.6%, *n* = 88) or high school (48.3%, *n* = 116).

### 2.2. Measures

All measures were self-reported, and data were collected through Qualtrics^TM^ (Qualtrics, Provo, Utah, UT, USA), an online survey platform. When necessary, scales were translated from English into Italian and back-translated by two independent researchers to assure linguistic validity. Some items were adapted to the adult context (e.g., items referring to the school environment were adapted to college and/or work situations). The order of the items within a scale was randomized, whereas the order of the scales was kept constant by the researchers. Cronbach’s alpha (α) or inter-item correlations (r) were employed to examine the internal consistency of the concepts (see Table 1). All the scales and items used in our analyses are available within the article and its supplementary information.

PSU was measured with the 10-item Smartphone Addiction Scale Short Version (SAS-SV) developed by Kwon and colleagues [81], translated and validated in Italian by De Pasquale, Sciacca and Hichy [82], and complemented with the item: “The first thing that I do in the morning is checking my smartphone (for example: checking notifications and social networks)”. Response options ranged from 1 “strongly disagree” to 6 “completely agree”. All items were averaged, with higher values indicating higher levels of PSU. Example items are: “Constantly checking my smartphone so as not to miss conversations between other people on Twitter or Facebook” and “The people around me tell me that I use my smartphone too much”.

Smartphone use was measured with an estimate of smartphone use [83,84] during a regular weekday and a regular weekend day with the following two open questions: “How many hours do you usually spend using your smartphone on a typical weekday?” and “How many hours do you usually spend using your smartphone on a typical weekend day?”. The final measure was obtained by averaging the two scores (ranging from a minimum of 1 h to a maximum of 9 h per day).

Social anxiety was measured with the 12-item Social Anxiety Scale for Adolescents (SAS-A [85]). Example items are: “I’m afraid that others will not like me”, “I get nervous when I meet new people”, and “I feel shy even with peers I know very well”. The validity, consistency, and test-retest reliability of the SAS-A have been tested in different populations [86,87]. Response options ranged from 1 “Not at all” to 5 “All the time”. All items were averaged, with higher scores indicating higher levels of social anxiety.

Dispositional trust was assessed with a single item, which has been used in prior studies and was shown to be a robust indicator [88,89,90]: “In general, how much do you trust people?”, rated on a Likert scale from 1 “Not at all” to 7 “Extremely”.

Covariates include gender (1 “male”, 2 “female”) and current occupation (1 “non-students”, 2 “students”).

### 2.3. Analytical Plan

Data analyses were conducted using SPSS Statistics v. 25, SPSS AMOS v. 23, the SPSS PROCESS macro by Hayes [91] and R statistical software [92]. SPSS was employed to check for normality of the data distributions, and for calculating descriptive statistics. Using the “pwr” package in R, power analysis resulted in 87.8% of power on the threshold of 80%, reflecting enough power to detect small-to-medium effects. The effect size was set to 0.2 according to literature describing social anxiety, smartphone use and problematic smartphone use as moderately correlated [27,45,93]. Pearson’s correlation and the point-biserial correlation were employed in a correlation matrix to determine the bivariate correlations among all the measures. Confirmatory factor analyses (CFA) were run with IBM SPSS AMOS 23, and Hu and Bentler’s [94] guidelines for various fit indices were used to determine the goodness of fit indexes. The following indexes and cut-off were considered: the chi-square (χ^2^) value and *p*-value, the comparative fit index (CFI; adequate if ≥0.90), the root mean square error of approximation (RMSEA; adequate if ≤0.08) and the standardized root mean square residual (SRMR; adequate if ≤0.08). The moderated mediation model was tested with manifest indicators by using model 59 in the SPSS PROCESS macro by Hayes [91]. The conditional indirect effects of the mediation were considered significant if the bootstrap confidence intervals for the index of the moderated mediation did not include the zero, as suggested by Hayes [95]. Moderation variables were mean-centered [96]. In order to test the interaction and obtain a meaningful and interpretable plot, raw regression weights were reported, as suggested by Whisman and McClelland [97]. Finally, one-way ANOVA tests were performed to plot the significant moderation effects considering the values of the predictor and moderator variables at the 16th, 50th and 84th percentiles, as suggested by Hayes [91]. The results were plotted using the Johnson-Neyman representation by the “rockchalk” package in R [98].

## 3. Results

### 3.1. Preliminary Results

At the univariate level, skewness and kurtosis values proved to be in the acceptable range between −1.96 and +1.96 (Skewness_MIN_ = −0.16—Skewness_MAX_ = 0.69; Kurtosis_MIN_ = −0.58—Kurtosis_MAX_ = 0.50) [99]. CFAs showed acceptable fit to the data for both the social anxiety (*χ*^2^ (51) = 163.787, *p* = 0.001, CFI = 0.929, RMSEA = 0.096, SRMR = 0.066) and the PSU scale (*χ*^2^ (41) = 105.866, *p* < 0.001, CFI = 0.908, RMSEA = 0.081, SRMR = 0.057).

Table 1 summarizes descriptive statistics for all averaged multi-item and single-item scales included in the final moderated mediation model alongside internal consistency measures and bivariate correlations among them.

Regarding the daily hours of smartphone use, 40% of the sample reported to use a smartphone for at least 4 h per day, while the median across the entire sample was 3.5 h per day.

As shown in Table 1, bivariate correlations revealed that social anxiety and smartphone use were significantly positively associated with PSU. Furthermore, females showed higher levels of social anxiety (M_female_ = 2.55, SD = 0.76, M_male_ = 2.35, SD = 0.74, t (238) = −2.09, *p* = 0.038) and smartphone use (M_female_ = 3.83, SD= 1.62, M_male_ = 3.25, SD = 1.42, t (238) = −2.94, *p* = 0.004) than males. Occupation status did not correlate significantly with any of the other variables. However, it was still taken into account as a covariate in the final model to control for possible biases due to work-related use of the smartphone. All significant correlation coefficients were below the threshold of 0.70, overcoming concerns of multicollinearity [100]. Moreover, the absence of significant correlations between dispositional trust and the other measures ensured its suitability as a moderator [101].

### 3.2. Primary Results

**Figure 2:**
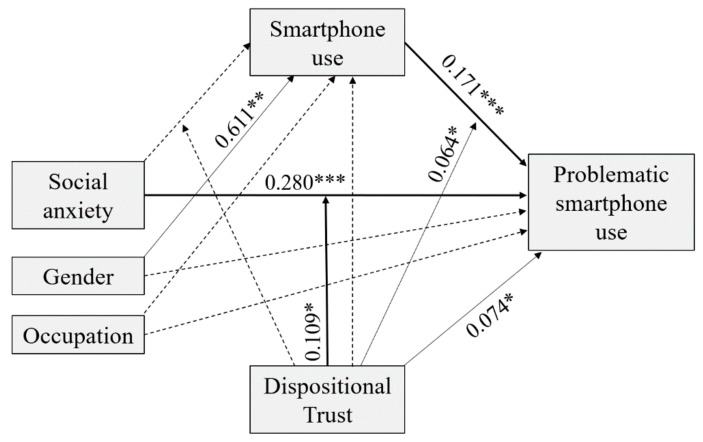
Results for the final moderated mediation model. Note: gender (1 = male, 2 = female) and current occupation (1 = non-students, 2 = students), * *p* < 0.05; ** *p* < 0.01; *** *p* < 0.001. Only significant path coefficients are displayed. Dotted lines display non-significant paths.

A moderated mediation model was performed to test the hypothesized relationships following a full information approach. A unique model was performed, as suggested by Shrout and Bolger [102] and Hayes [91], who argue that indirect effects can be significant regardless of the significance of single mediation paths. The results are shown in Figure 2 and summarized in Table 2.

Firstly, we hypothesized that higher levels of social anxiety are associated with higher levels of PSU. The direct path between social anxiety and PSU was positive and significant (B = 0.280, *p* = 0.001). Thus, H1 was supported. Secondly, we hypothesized that social anxiety is positively associated with smartphone use. However, the relationship between the two concepts was non-significant (B = −0.152, *p* = 0.255). Hence, H2 was not supported. Thirdly, we hypothesized that higher levels of smartphone use are related to higher levels of PSU. The direct path between the two concepts was positive and significant (B = 0.177, *p* < 0.001). Thus, H3 was supported. Consequently, the indirect relationship between social anxiety and PSU, mediated through smartphone use, was also non-significant. For the concerns of our covariates, smartphone use was only predicted by gender (B = 0.611, *p* = 0.003), with females reporting higher levels of smartphone use.

**Figure 3:**
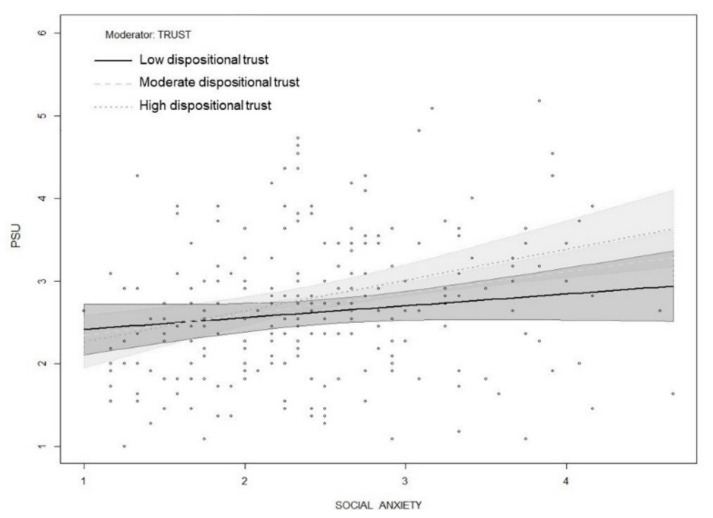
Interaction between social anxiety and dispositional trust on PSU.

**Figure 4:**
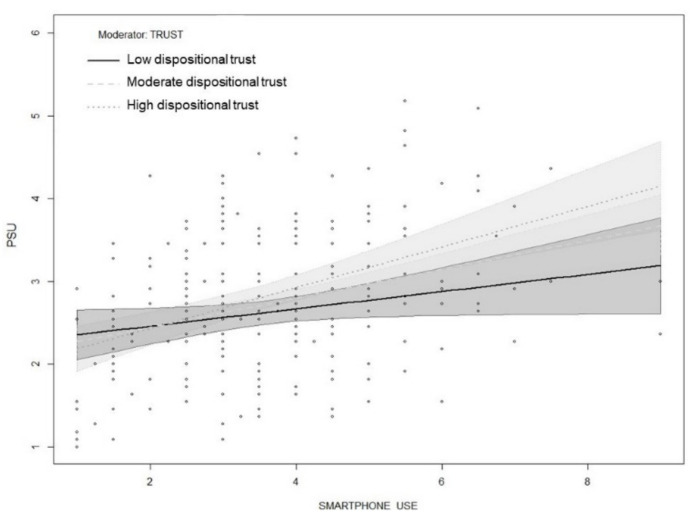
Interaction between smartphone use and dispositional trust on PSU.

Finally, we hypothesized that dispositional trust moderates the relationship between social anxiety, smartphone use, and PSU. Evaluation of the interaction terms revealed that dispositional trust did not moderate the relationship between social anxiety and smartphone use. However, dispositional trust moderated the direct relationships between social anxiety and PSU as well as smartphone use and PSU. Specifically, the conditional direct effect of social anxiety on PSU was positive and significant at moderate (B = 0.261, *p* < 0.001) and high (B = 0.418, *p* < 0.001) levels of dispositional trust, where moderate and high were at the 50th and 84th percentiles of the dispositional trust mean-centered distribution, respectively. Similarly, the direct conditional effect of smartphone use on PSU was positive and significant at moderate (B = 0.166, *p* < 0.001) and high (B = 0.258, *p* < 0.001) levels of dispositional trust. These results partially supported H4. The overall moderated mediation model explained 22% of the variance in PSU. In order to visualize the conditional effect of three levels of dispositional trust (low, moderate, high) on the relationship between social anxiety and PSU as well as smartphone use and PSU, the interaction effects are shown in Figure 3 and Figure 4.

## 4. Discussion

Smartphones have become an indispensable part of people’s everyday life, especially among young adults. The pervasiveness of the smartphone is reinforced by its portability and utility for both social [103] and non-social purposes [63]. This poses questions on whether dispositional and behavioral predispositions are risk factors of problematic smartphone use (PSU), which has been linked to negative health outcomes, with prevalence rates continuing to augment [104]. Considering theoretical insights from the Brand and colleagues’ I-PACE model [25] and Elhai and colleagues’ model [29] on the link between anxiety and problematic smartphone use, we aimed to examined a moderated mediation model predicting PSU from social anxiety, partially mediated through smartphone use, and moderated by dispositional trust.

Our results were consistent with previous research, sustaining the direct link between social anxiety and PSU [40,47,105]. Indeed, according to Brand and colleagues’ [25], socially anxious individuals proved to be more at risk of developing a problematic attachment to the smartphone due to the easy access to online social gratifying contents as well as their preference for online interactions [12,93]. That said, socially anxious individuals are more likely to find relief from interactions carried out via the smartphone, which allows them to avoid potentially stressful and face-threatening interpersonal interactions, due to reduced dependence on non-verbal cues and an augmented possibility to control the social situation. The preference for online interactions may lead to a dysfunctional attachment to the device itself, i.e., PSU [106]. In addition, excessive reassurance-seeking behaviors, frequent in socially anxious individuals, may trigger a habitual and constant checking of the smartphone, looking for social-related notifications [50]. At the same time, socially anxious people may show higher levels of PSU through a non-social smartphone use, e.g., by searching for information, entertainment and relaxation activities, all described as media-related predictors of PSU [27,107,108]. By doing so, socially anxious people consume non-social contents to fill their time, obtain gratifications, escape from problems, and compensate for the lack of face-to-face interactions [63,107]. In general, the significant relationship between social anxiety and PSU is also in line with the results of contemporary meta-analyses [30,31,56], reporting a large correlational effect size between the two concepts.

Although it was associated with PSU, social anxiety was not associated with smartphone use in terms of the time spent on the device on a typical day. Although previous studies reported higher levels of anxiety among heavy smartphone users [39,57,58], an explanation for the non-significant relationship between social anxiety and smartphone use may be that a longer duration of smartphone use in young adults may likely be due to work and study-related use, and other activities such as gaming, online searches for information, and organization of day-to-day activities, which do not necessarily involve online social interactions linked to social anxiety.

In other words, the amount of time spent on the device is not a partial mediator in the relationship between social anxiety and PSU. This finding contradicts Elhai and colleagues’ model [29] linking anxiety and problematic smartphone use indirectly through higher levels of smartphone use. However, it should be noted that the model considers frequency of smartphone use while we considered duration of use in the present study. Both are highly related but distinct measures of smartphone use, with frequency, i.e., high intensity of repeated checking behavior, being a stronger predictor of PSU than duration of device use [21,109]. Thus, future research should consider the frequency of smartphone use and combine it with automatically recorded smartphone use data to provide additional valuable insights on the mediating role of smartphone use in the relationship between social anxiety and PSU [109,110].

An additional and innovative result of the present study is the partially moderating role of dispositional trust in the relationship between social anxiety and PSU as well as smartphone use and PSU. More precisely, our results suggest that those who spent more time on the device and trusted others more showed higher levels of PSU. Young individuals with high levels of trust have been found to be less adapted in their social context, compared to people with intermediate levels of trust [111,112,113]. Highly trusting in others has also been described as having its “dark side” [114], because some trust experiences may result in an unwelcome burden due to less-than-benign intent in the people involved. In general, individuals with high levels of dispositional trust have been described as more prone to look for online interactions, initiate smartphone-based communications [75], and invest their time in the online social context [61,74]. As more and more connections are initiated and maintained through social media, or via smartphone-mediated communication in general, this predisposition may strengthen PSU. This is particularly true for socially anxious individuals who tend to avoid face-to-face interactions [106], and for those particularly attached to their smartphones in terms of usage and time engaging in smartphone activities.

Finally, consistent with previous scientific literature [6,57], the results of our study showed higher levels of smartphone use in females compared to males. However, gender did not significantly predict PSU. This finding, although not of focal interest in this study, stands in contrast to results from previous studies identifying females to be at higher risk of developing a problematic attachment to their smartphones [23].

This study has several limitations that should be acknowledged. Firstly, the collected data were cross-sectional and did not allow any inference on causal effects among the tested relationships. A longitudinal design would provide insights on the stability of the concepts over time and their bidirectional effects (e.g., using a cross-lagged or, ideally, a random-intercept cross-lagged panel model). Secondly, all measures were self-reported, raising the concern of social desirability, recall, and estimation biases. The self-assessments of smartphone use, especially, may suffer from time distortions, generating cognitive biases [21,115,116]. Finally, future research should also include other personality traits such as gratification and impulse control concepts as influential aspects which may transform smartphone use in compulsive behavior [25,117,118].

Despite these shortcomings, the present study provides evidence that social anxiety and smartphone use, as well as their interaction with dispositional trust, are directly associated with PSU among young adults, which highlights the role of psychological, social, and behavioral risk factors of this new form of problematic behavior.

## 5. Conclusions

Heavy smartphone users, with the tendency of trusting others, are more at risk of PSU, which can be explained by their continuous search for connections. Conversely, lower levels of dispositional trust toward others do not boost socially anxious and heavy users to developing PSU [119]. The link between smartphone use and the dark side of dispositional trust would benefit from further research on a content- and functionality-specific use of the smartphone, as suggested by Brand and colleagues [25] to identify PSU and its psychological determinants.

## Figures and Tables

**Table 1:** Means, standard deviations, internal consistency measures and bivariate correlations (*n* = 240).

Measures	M (SD)	α/r	1	2	3	4	5
1. Social anxiety	2.45 (0.75)	0.891	1				
2. Dispositional trust	4.17 (1.34)	-	−0.034	1			
3. Smartphone use	3.54 (1.55)	0.572	−0.046	−0.030	1		
4. Problematic smartphone use (PSU)	2.71 (0.85)	0.822	0.218 **	0.101	0.329 ***	1	
5. Gender ^a^		-	0.134 *	−0.047	0.187 **	0.121	1
6. Occupation ^a^		-	0.106	−0.083	−0.007	−0.045	0.095

* 0.05 level (2-tailed). ** 0.01 level (2-tailed). *** 0.001 (2-tailed). ^a^ point-biserial correlations between gender (1 = male, 2 = female) and current occupation (1 = non-students, 2 = students) and other variables are point-biserial.

**Table 2:** Regression results for the moderated mediation model.

Predictors	Smartphone Use			Problematic Smartphone Use		
	B	SE	[95% CI]	B	SE	[95% CI]
Constant	−0.890	0.446	[−1.77; −0.12]	2.782 ***	0.225	[2.34; 3.22]
Gender	0.611 **	0.201	[0.22;1.01]	0.066	0.102	[−0.13; 0.27]
Occupation	0.017	0.207	[−0.43; 0.39]	−0.099	0.104	[−0.30; 0.11]
Social anxiety	−0.152	0.134	[−0.42; 0.11]	0.280 ***	0.067	[0.15; 0.41]
Dispositional trust	−0.028	0.075	[−0.17; 0.12]	0.074 *	0.037	[0.00; 0.15]
Dispositional trust + Social anxiety	−0.033	0.091	[−0.21; 0.15]	0.109 *	0.046	[0.02; 0.20]
Smartphone use				0.177 ***	0.033	[0.11; 0.24]
Dispositional trust + Smartphone use				0.064 **	0.028	[0.01; 0.12]
F	(5, 234) = 2.02			(7, 232) = 9.00 ***		
*R* ^2^	0.04			0.21		

Note: SE, standard error; 95% CI, confidence intervals with lower and upper limits. Gender 1 = male, 2 = female; current occupation 1 = non-student, 2 = student; * *p* < 0.05; ** *p* < 0.01; *** *p* < 0.001.

## Data Availability

The data presented in this study are openly available at https://forsbase.unil.ch (accessed on 19 January 2021), reference number 13984.
